# Morphological and molecular characterization of two species of *Neothada* Khan, 1973 (Nematoda: Tylenchidae) from Iran, with notes on *N. cancellata*

**DOI:** 10.21307/jofnem-2020-131

**Published:** 2021-02-02

**Authors:** Manouchehr Hosseinvand, Ali Eskandari, Reza Ghaderi

**Affiliations:** 1 Department of Plant Protection, Faculty of Agriculture, University of Zanjan, 45371-38791, Zanjan, Iran; 2 Department of Plant Protection, School of Agriculture, Shiraz University, 71441-65186, Shiraz, Iran

**Keywords:** *Neothada cancellata*, *Neothada hades*, *Neothada major*, 28 S rDNA, Khuzestan, phylogeny, Bayesian inference

## Abstract

Three species of the genus *Neothada*, including *N. cancellata, N. hades* and *N. major*, collected from the rhizosphere of mosses in Khuzestan, southwestern Iran, are redescribed and illustrated. *Neothada hades* and *N*. *major* are new records from Iran. *Neothada hades* has 14 longitudinal incisures excluding the lateral field, body length of 586 (505–674) µm, stylet 10.5 (10.0–10.8) µm in length bearing distinct basal knobs, and an elongated-conical tail 70.4 (65–74) µm long with a finely to bluntly rounded terminus. *N. major* possesses 18–20 longitudinal incisures excluding the lateral field, body length of 657 (600–728) µm, stylet 10.9 (10.3–11.7) µm long with basal swellings but not distinct knobs, and an elongated-conical tail 78.2 (70–83) µm long ending to a finely to bluntly rounded terminus. Molecular phylogenetic studies of the two species (*N. hades* and *N. major*) with 664 bp of D2-D3 expansion segments of 28 S rDNA revealed that they form a clade with *N*. *cancellata*.

Khan (1973) proposed the genus *Neothada* to accommodate *Thada cancellata* Thorne, 1941 and *T. tatra* Thorne & Malek, 1968, two species with longitudinal incisures around the circumference of the body in addition to the lateral fields. Siddiqi (2000) accommodated the genera *Neothada* and *Thada* in the subfamily Thadinae Siddiqi, 1986, but Geraert (2008) placed them in the subfamily Boleodorinae Khan, 1964. According to Geraert (2008), the genus *Neothada* currently contains six valid species namely: *N. tatra* (Thorne and Malek, 1986) Khan, 1973 as the type species; *N. cancellata* (Thorne, 1941) Khan, 1973; *N. costata* (Geraert and Raski, 1986) Siddiqi, 2000; *N. geraerti* (Andrássy, 1982) Siddiqi, 1986; *N. hades* Heyns & van den Berg, 1996 and *N. major* Maqbool & Shahina, 1989. So far, only *N. cancellata* has been reported from Iran (Ghorbanzad et al., 2012; Yaghoubi et al., 2015). *Neothada cancellata*, *N. hades* and *N. major* were all collected in Khuzestan province, Iran and compared. The latter two species are illustrated and described by morphological, morphometric and molecular approaches.

## Materials and methods

### Morphological characterization

Soil samples were collected from the rhizosphere of mosses, *Cynodon dactylon* (L.) Pers. and *Prosopis cineraria* (L.) Druce, in Dezful and Bostan, both cities in Khuzestan province, southwestern Iran. Nematodes were extracted with the tray method (Whitehead and Hemming, 1965), killed and fixed in hot FPG (4:1:1, formaldehyde: propionic acid: glycerol), processed to anhydrous glycerol and mounted in glycerol on permanent slides (De Grisse, 1969). Specimens were studied with a light microscope equipped with a Dino-eye microscope eye-piece camera and Dino Capture version 2.0 software. Specimens were identified to species with the identification key of Geraert (2008).

### DNA extraction, PCR and sequencing

Nematode DNA was extracted from single individuals and DNA extracts were stored at −20°C until use as PCR template. Protocols for DNA extraction were followed as described by Tanha Maafi et al. (2003). Fragments of D2-D3 expansion segments of 28 S rDNA were amplified using the forward D2A (5’–ACAAGTACCGTGAGGGAAAGT–3’) and reverse D3B (5’–TCGGAAGGAACCAGCTACTA–3’) primers (Nunn, 1992). The 30 μl PCR contained 15 μl Taq DNA Polymerase 2 × MasterMix (Ampliqon, Denmark), 1 μl (10 pmol μl^−1^) each of forward and reverse primers, 2 μl of DNA template and 11 μl deionised water. This mixture was placed into a Hybaid Express thermal cycler (Hybaid, Ashford, Middlesex, UK). The thermal cycling profile was denaturation at 95°C for 4 minutes, then 33 cycles of denaturation at 94°C for 30 seconds, annealing at 57°C for 30 seconds, and extension at 72°C for 90 seconds. A final extension was performed at 72°C for 10 minutes. The quality of PCR was checked by electrophoresis of 4 μl of the PCR reaction in 1% agarose gel containing ethidium bromide. Products were visualized and photographed under UV light. The length and concentration of each PCR product was measured by comparison with a low DNA mass ladder (Invitrogen, Carlsbad, CA). The PCR product was purified and sequenced directly for both strands using the same primers with an ABI 3730XL sequencer (Bioneer, Seoul, South Korea). The newly obtained sequences were submitted to GenBank database under accession numbers MN970001 and MN970002 for the D2-D3 expansion fragments of 28 S sequences.

### Phylogenetic analyses

For phylogenetic relationships, analyses were based on D2-D3 expansion fragment of 28 S rDNA. The newly obtained sequences were edited and aligned with other sequences available in GenBank using the Muscle alignment tool implemented in MEGA7 (Kumar et al., 2016). The ambiguously aligned parts and divergent regions were identified using the online version of Gblocks 0.91b (Castresana, 2000) and were removed from the alignments with MEGA7. The best-fit model of nucleotide substitution used for the phylogenetic analysis was statistically selected using jModelTest 2.1.10 (Darriba et al., 2012). Phylogenetic tree was generated with a Bayesian inference method using MrBayes 3.2.6 (Huelsenbeck and Ronquist, 2001; Ronquist et al., 2012). *Aphelenchus avenae* Bastian, 1865 (KP527123) was chosen as outgroup for the tree according to (Bai et al., 2020; Hosseinvand et al., 2020b; Yaghoubi et al., 2015). The analysis under general time-reversible model of sequence evolution with correction for invariable sites and a gamma-shaped distribution (GTR + I + G) model was initiated with a random starting tree and run with the Markov Chain Monte Carlo (MCMC) for 1  ×  10^6^ generations. The tree was visualized and saved with FigTree 1.4.3 (Rambaut, 2016) and edited with Adobe® Acrobat® XI Pro 11.0.1.

## Results

### Systematics

**Figure 1:**
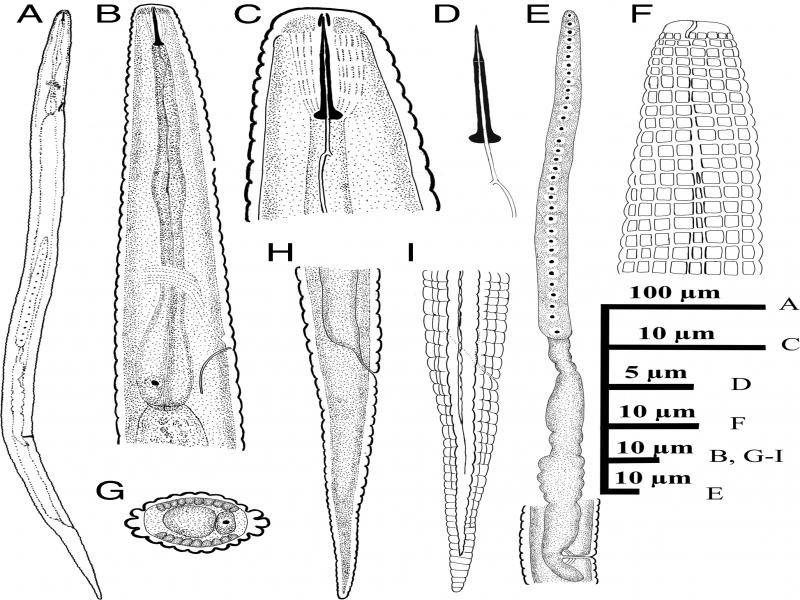
Iranian population of *Neothada hades*. Female (A-I): (A) Entire body; (B) Anterior end and pharyngeal region; (C) Lip region and stylet; (D) Stylet; (E) Reproductive system; (F) Amphidial aperture; (G) Cross section from mid-body; (H-I) Posterior end.

**Figure 2:**
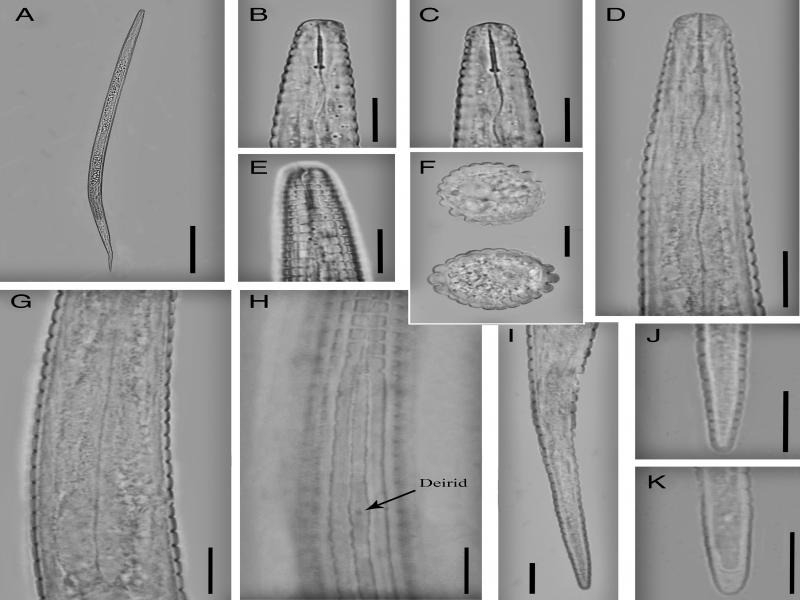
Iranian population of *Neothada hades.* Female (A-K): (A) Entire body; (B-D) Lip region and stylet; (E) Amphidial aperture; (F) Cross section from mid-body; (G) Basal pharyngeal bulb and secretory-excretory pore; (H) Deirid and lateral field; (I-K) Posterior end. (Scale bars: A = 100 µm; B-K = 10 µm).

*Neothada hades* Heyns & Van den Berg, 1996 (Figures 1 and 2; Table 1).

**Table 1.** Morphometric data of *Neothada major*, *N. hades* and *N. cancellata* from Iran.

	Neothada major			N. hades			N. cancellata
	Present study		Maqbool and Shahina (1989)	Present study		Heyns and Van den Berg (1996)	Present study
n	15	cv	15	10	cv	10	8
L	657±40.6 (600–728)	6.1	640–800	586±50.3 (505–674)	8.5	530–620	561±32.5 (501–596)
L'	579±37.8 (521–645)	6.5	–	515±48.1 (440–600)	9.3	–	491±30.9 (431–527)
Head-Vulva	461±27.2 (420–508)	5.9	–	414±35.5 (354–467)	8.5	–	396±24.4 (349–424)
R Head-Vulva	142±11.7 (120–160)	8.2	154–156	147±8.3 (127–157)	5.6	–	146±9.9 (125–156)
R Head-Anus	174±12.4 (149–194)	7.1	–	175±9.2 (152–185)	5.2	–	173± 11.1 (150–185)
Stylet	10.9±0.3 (10.3–11.7)	3.3	12–14.4	10.5±0.2 (10.0–10.8)	2.1	9.0–10.5	10.8±0.2 (10.6–11.2)
a	35.9±4.1 (29–44)	11.4	34–39	30.3±2.0 (27.0–33.7)	6.8	25–31	29.5±1.7 (27–32)
b	5.7±0.4 (5.2–6.5)	6.9	5.9–6.3	5.3±0.2 (4.8–5.7)	5.4	5.3–6.4	5.2±0.2 (4.7–5.5)
c	8.3±0.3 (7.5–8.7)	3.7	9.0–10.2	8.3±0.4 (7.5–9.1)	5.8	8.1–10.3	8.0±0.3 (7.5–8.6)
c'	6.4±0.3 (5.6–7.1)	6.1	5.5–6.0	6.0±0.3 (5.5–6.5)	5.8	4.6–6.0	6.1±0.4 (5.5–6.6)
V	70.2±0.7 (68.8–71.2)	1.0	70–73	70.6±1.1 (68.5–72.7)	1.6	70–74	70.9 ± 0.6 (70.0–71.8)
V'	79.7±0.9 (78.0–81.1)	1.1	–	80.3±1.3 (77.0–81.9)	1.6	–	80.8±0.5 (80.4–82.4)
R	202±12.5 (175–222)	6.2	215–245	204±10.1 (179–216)	4.9	149–160	189± 12.3 (175–191)
Excretory pore	97.2±8.7 (75–110)	9.0	–	94.7±5.8 (87–104)	6.1	–	92.5±6.7 (86–100)
Pharynx	114±7.6 (94–127)	6.7	108–133	109.5±8.4 (95–119)	7.7	–	107±9.3 (90–110)
R Pharynx	41±3.0 (36–50)	7.2	45–50	47±3.1 (43–52)	6.6	30–38	45±2.5 (42–50)
Annulus width	3.8±0.7 (3.2–5.6)	20.0	3.2–4.0	2.9±0.2 (2.7–3.5)	8.3	4.0–4.6	3.0±0.2 (2.6–3.6)
Body width	18.4±1.7 (15.6–21.0)	9.6	–	19.2±0.8 (17.8–21.0)	4.5	–	18.8±0.6 (18–20)
Vulva body width	17.5±1.5 (15–20)	9.0	–	17.9±0.7 (16.5–19.0)	4.0	–	17.5±0.7 (15.5–18.8)
Vulva-Anus	117±11.8 (101–137)	10.0	–	101.6±14.9 (86–138)	14.7		95±7.1 (81–103)
R Vulva-Anus	32±3.8 (28–42)	11.8	36–40	27±1.4 (25–29)	5.4	21–26	27±1.5 (25–28)
Tail/Vulva-Anus	0.6±0.1 (0.6–0.7)	7.7	–	0.7±0.1 (0.5–0.7)	10.1	–	0.7±0.1 (0.6–0.7)
Anal body width	12.2±0.9 (10.8–14.0)	7.3	–	11.5±0.5 (10.8–12.3)	4.7	–	11.1±0.5 (10.5–12.0)
Tail length	78.2±3.5 (70–83)	4.5	67–80	70.4±2.7 (65–74)	3.9	55–66	69±2.4 (65–71)

Note: All measurements are in μm and in the form: mean ± standard deviation (range).

### Description

#### Female

Body straight to slightly ventrally curved. Cuticular annules prominent, at neck 1.5–2.2 µm and at mid-body 2.7–3.5 µm in width. Lateral field with four incisures, delimiting three ridges, starting from middle of procorpus and continue to five or seven annules anterior to tail tip. In addition to the lateral lines, 14 evenly spaced longitudinal incisures around the circumference of the body. Lateral field 6.0–8.5 µm wide occupying 32–42% of the corresponding body diameter, its ridges more pronounced and larger than other longitudinal incisures; annulation and longitudinal incisures produce rectangular tessellation. Cephalic region flatly rounded, with two annules, 7.2–7.5 µm wide at base and 2.5–3.0 µm high. Amphidial aperture a conspicuous longitudinal to slightly bent slit extending as far as the second neck annule. Cephalic framework inconspicuous, weakly sclerotized. Stylet delicate, conus length about one-third (3.2–3.9 µm, or 31–36%) of the total stylet length, 7–8 annules from anterior end; knobs conspicuous, rounded, 1.7–2.2 µm wide. Dorsal pharyngeal gland opening 2.6–4.0 µm from stylet base. Corpus cylindroid with slightly swollen median bulb lacking valve; isthmus as wide as procorpus, nerve ring at mid-isthmus and located 56–73 µm from anterior end. Pharyngeal basal bulb short, pyriform, 7.5–9.0 µm wide, 19–24 µm in length. Pharyngo-intestinal valve hemispherical. Excretory pore slightly sclerotized, at middle of basal bulb, 38–45 annules from anterior end. Hemizonid one annule anterior to the excretory pore, 85–104 µm from anterior end. Deirids adjacent to the level of excretory pore, 87–107 µm from anterior end. Vulva a transverse slit, not protruding, without lateral flaps. Vagina width 6.7–8.0 µm, 37–46% of vulva body diameter. Post-vulval uterine sac length 9.0–12.8 µm or 57–63% of vulval body diameter. Spermatheca long, variable in shape, near-rectangular, 7.5– to 9.0 µm × 26 to 33 µm. Ovary outstretched, oocytes arranged in a single row. Rectum curved to slightly sigmoid, length half of anal body diameter. Tail elongate-conoid, tail tip finely to bluntly rounded, 27–33 annules on ventral side of the tail.

#### Male

Not found.

#### Voucher specimens

In all, 10 females are deposited in the nematode collection of the Department of Plant Protection, Faculty of Agriculture, University of Zanjan, Zanjan, Iran.

#### Habitat and locality

Soil around of mosses in Dezful, Khuzestan Province, southwestern Iran, by Manouchehr Hosseinvand at February 2017 (GPS coordinates: 48°47‘18“N, 26°36‘32“E).

#### Morphological remarks

*Neothada hades* can be distinguished from all other known species of the genus by possession of distinct stylet knobs. The morphology and morphometrics of the Iranian population are coincident with the original species description of Heyns & Van den Berg (1996), except for the number and width of body annules (179–216 vs 149–160 and 2.7–3.9 vs 4.0–4.6 µm, respectively), tail length (65–74 vs 55–66 µm) and absence of males (vs presence).

**Figure 3:**
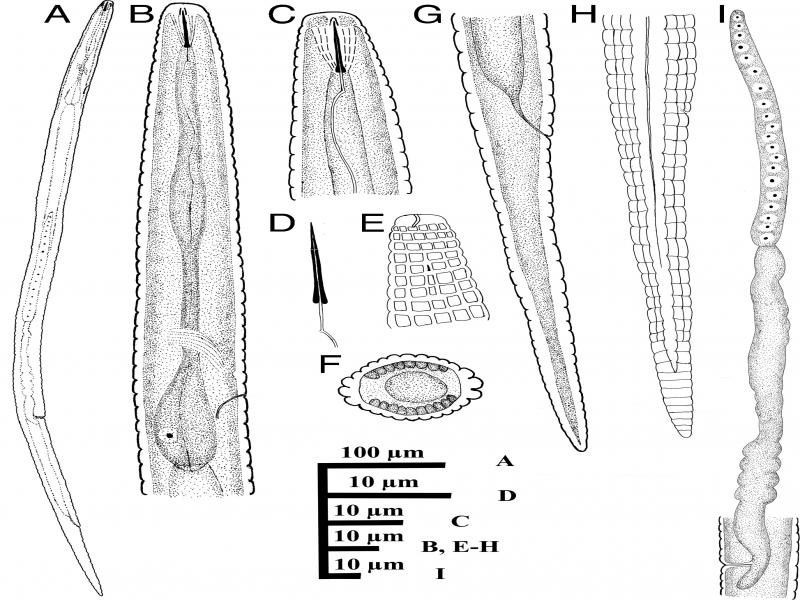
Iranian population of *Neothada major.* Female (A-I): (A) Entire body; (B) Anterior end and pharynx; (C) Lip region; (D) Stylet; (E) Amphidial aperture; (F) Cross section from mid-body; (G-H) Posterior end; (I) Reproductive system.

**Figure 4:**
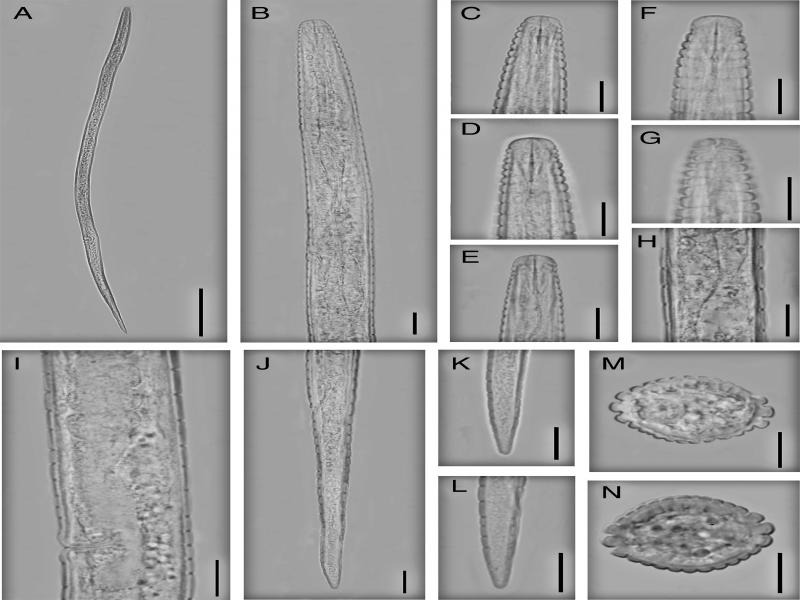
Iranian population of *Neothada major.* Female (A-N): (A) Entire body; (B) Anterior end and pharyngeal region; (C-F) Lip region and stylet; (G) Amphidial aperture; (H) Annules at mid-body; (I) Vulval region; (J-L) Posterior end; (M, N) Cross section from mid-body. (Scale bars: A = 100 µm; B-N = 10 µm).

*Neothada major* Maqbool & Shahina, 1989 (Figures 3 and 4; Table 1).

### Description

#### Female

Body straight to slightly ventrally curved in posterior half. Cuticular annules prominent, at neck 1.5–2.2 µm and at mid body 3.2–5.6 µm in width. Lateral field with four incisures, delimiting three ridges, starting at middle of isthmus and continue to seven or nine annules anterior to tail tip. In addition to the lateral lines, 19–20 evenly spaced longitudinal incisures around the circumference of the body. Lateral field 5.5–8.0 µm wide or occupying 30–39% of corresponding body diameter; annulation and longitudinal incisures produce rectangular tessellation. Cephalic region flatly rounded, with one or two annules, 6.8–8.1µm wide at base and 2.5–3.6 µm high. Amphidial aperture a conspicuous longitudinal to slightly bent slit, extending as far as the second neck annule. Cephalic framework inconspicuous, weakly sclerotized. Stylet delicate, conus length about one-third, 3.4–3.9 µm or 32–35% of the total stylet length, without knobs but slightly swellings, 7–8 annules from anterior end. Dorsal pharyngeal gland opening 3.0–4.0 µm from stylet base. Corpus cylindroid, median bulb weakly developed, lacking valve; isthmus slender, slightly narrower than procorpus, nerve ring at posterior part of isthmus and located at 66–86 µm from anterior end. Basal bulb short, pyriform to slightly saccate, 7.7–10.0 µm wide and 18–29 µm in length. Pharyngo-intestinal valve hemispherical. Excretory pore at anterior part of basal bulb, 35–40 annules from anterior end. Hemizonid one to two annules anterior to the excretory pore, 88–104 µm from anterior end. Deirid at level of excretory pore, 92–113 µm from anterior end. Vulva a transverse slit, not protruding, without flaps. Vagina 6.2–9.0 µm or 40–54% of vulva body diameter. Post-vulval uterine sac length 9.0–10.0 µm or 55–74% of vulva body diameter. Spermatheca very long, without specific shape, without sperm. Ovary outstretched, oocytes in single row. Rectum curved to slightly sigmoid, length half of anal body diameter. Tail elongate-conoid, tail tip with narrowly rounded tip, 25–30 annules on ventral side of the tail.

#### Male

Not found.

#### Voucher specimens

In all, 15 females are deposited in the nematode collection of the Department of Plant Protection, Faculty of Agriculture, University of Zanjan, Zanjan, Iran.

### Habitat and locality

Soil around *Prosopis cineraria* (L.) (Jand) in Bostan, Khuzestan Province, southwestern Iran, by Manouchehr Hosseinvand, March 2019 (GPS coordinates: 48°05‘19“N, 43°45‘31“E).

### Morphological remarks

The morphology and morphometrics of the Iranian population agree well with the type population of *N. major*, except for minor differences in stylet length (10.3–11.7 vs 12–14.4), c ratio (7.5–8.7 vs 9.0–10.2) and absence of males (vs presence).

**Figure 5:**
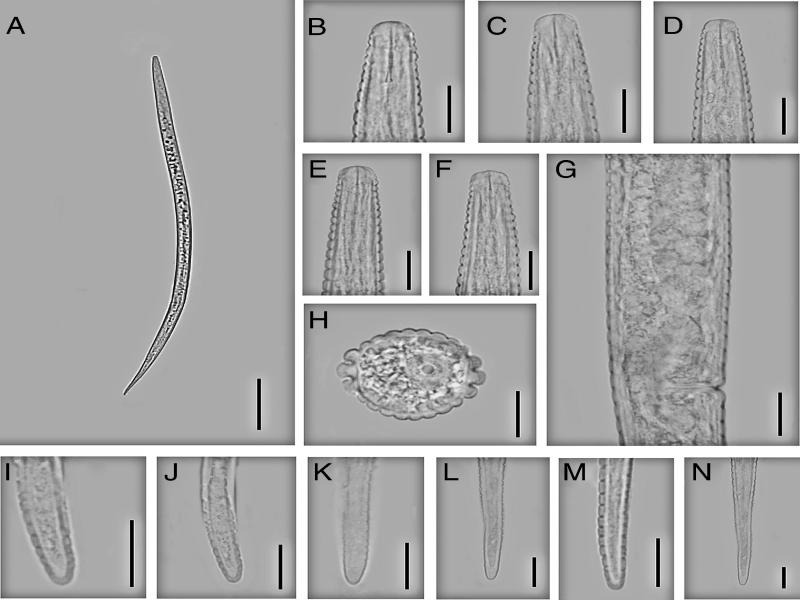
Iranian population of *Neothada cancellata*. Female (A-N): (A) Entire body; (B-F) Anterior end and stylet; (G) Vulval region; (H) Cross section from mid-body; (I-N) Posterior end.

*Neothada cancellata* (Thorne, 1941) Khan, 1973 (Figure 5; Table 1).

### Description

#### Female

Body straight to slightly ventrally curved. Cuticle annules prominent at mid body 2.6–3.6 µm. Lateral field with four incisures, delimiting three ridges, starting at mid of procorpus to five or seven annules anterior to tail tip. In addition to the lateral lines, 14–16 evenly spaced longitudinal incisures around the circumference of the body. Head flatly rounded, with two annules. Amphidial aperture a conspicuous longitudinal to slightly bent slit, extending as far as the second neck annule. Cephalic framework inconspicuous, weakly sclerotized. Stylet delicate, conus length about one-third of the total stylet length. Dorsal pharyngeal gland opening 2.5–4.0 µm from stylet base. Median bulb lacking valve, isthmus as wide as procorpus, nerve ring at mid of isthmus, basal bulb short, pyriform. Excretory pore slightly sclerotized, at mid of basal bulb. Deirids at level of excretory pore. Vulva with transverse slit. Vagina length less than half of vulva body diameter. Post-vulval uterine sac 10–13 µm. Spermatheca long, variable in shape. Ovary outstretched, oocytes in single row. Tail elongate-conoid, tail tip bluntly rounded, 26–33 annules on ventral side of tail.

#### Male

Not found.

#### Morphological remarks

The morphology and morphometrics of the Iranian population agree completely with the data given by Geraert (2008). The present population is different from the other population of Iran (Yaghoubi et al., 2015) in body length (501–596 vs 573–668 µm), number of body annules (175–191 vs 143–160), width of body annules (2.6–3.6 vs *ca* 5.0 µm), *c* ratio (7.5–8.6 vs 8.8–11.0), V (70–71.8 vs 72.8–75.6) and absence of males (vs presence).

#### Habitat and locality

Soil around roots of *Cynodon dactylon* (L.) Pers. and mosses in Dezful, Khuzestan Province, southwestern Iran, by Manouchehr Hosseinvand at February 2017 (GPS coordinates: 48°47‘21“N, 26°35‘06“E).

### Molecular phylogenetic status

**Figure 6:**
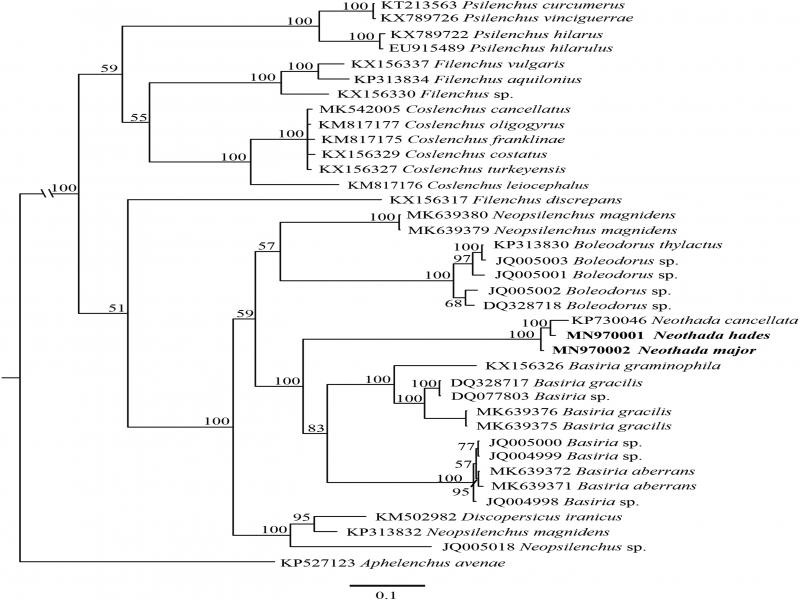
Bayesian Inference analysis tree under general time-reversible model of sequence evolution with correction for invariable sites and a gamma-shaped distribution model (GTR + I + G) from known and the newly sequenced *Neothada hades* and *N. major* based on sequences of the D2-D3 expansion fragments of the 28 S rDNA region. Bayesian posterior probabilities (50%) are given for each clade. Scale bar shows the number of substitutions per site.

Two new D2-D3 28 S rDNA gene sequences were obtained in the present study (MN970001 and MN970002). These sequences showed a 96–97% similarity values to *N. cancellata* (KP730046), and 80–82% similarity with *Basiria* spp. using BlastN search in NCBI. The partial D2-D3 of 28 S rDNA gene sequences alignment contained 38 taxa including *Aphelenchus avenae* (KP527123) as outgroup taxon and was 664 bp in length after removing ambiguously aligned regions. The 50% majority rule consensus phylogenetic tree generated from the partial D2-D3 region of 28 S rDNA alignment by Bayesian inference (BI) analysis under GTR + I + G model is presented in Figure 6.

*N. hades* is closely related to *N. cancellata* (PP = 100) and both species are related in the clade with *N. major* (PP = 100). *N. hades* differs from *N. cancellata* by 16 bp (2.1%) and from *N. major* by 11 bp (1.6%), and *N. major* differs from *N. cancellata* by 20 bp (1.9%).

## Discussion

In our 28 S rDNA phylogeny, *Neothada* is highly supported as a valid genus (Figure 6), similar to previously published works (Yaghubi et al., 2015; Hosseinvand et al., 2020a), and as a member of the subfamily Boleodorinae as suggested by Geraert (2008). In this tree, *Neothada* formed a sister clade with species of *Basiria* Siddiqi, 1959, the other representative of the subfamily Boleodorinae (PP = 100); *Boleodorus* and two sequences of *Neopsilenchus magnidens* (MK639379, MK639380) formed a basal clade with them. *Discopersicus iranicus* (KM502982) and two sequences of *Neopsilenchus* (KP313832, JQ005018), other likely members of the subfamily Boleodorinae, are placed in other clades in the tree. According to Hosseinvand et al. (2020c), *Thada* forms a clade with *Filenchus* Andrássy, 1954 in the 18 S rDNA tree, which is not in agreement with the position of *Thada* along with *Neothada* in the subfamily Boleodorinae. We believe that in the subfamily Boleodorinae, the distance of dorsal esophageal gland orifice (DGO) from the stylet base and development of median bulb and stylet knobs are also important diagnostic characters as much as the shape of amphidial apertures. In the genus *Neothada*, the number of longitudinal incisures, degree of stylet knobs development and tail tip shape are important diagnostic characters for identification of the known species.
